# Modeling of the jasmonate signaling pathway in *Arabidopsis thaliana* with respect to pathophysiology of *Alternaria* blight in *Brassica*

**DOI:** 10.1038/s41598-017-16884-3

**Published:** 2017-12-01

**Authors:** Rajesh Kumar Pathak, Mamta Baunthiyal, Neetesh Pandey, Dinesh Pandey, Anil Kumar

**Affiliations:** 1 Department of Biotechnology, Govind Ballabh Pant Institute of Engineering & Technology, Pauri Garhwal, 246194 Uttarakhand India; 2 Centre for Agricultural Bioinformatics, ICAR-Indian Agricultural Statistics Research Institute (IASRI), Pusa, 110012 New Delhi India; 3 Department of Molecular Biology & Genetic Engineering, College of Basic Sciences & Humanities, G. B. Pant University of Agriculture & Technology, Pantnagar, 263145 India

## Abstract

The productivity of Oilseed *Brassica*, one of the economically important crops of India, is seriously affected by the disease, *Alternaria* blight. The disease is mainly caused by two major necrotrophic fungi, *Alternaria brassicae* and *Alternaria brassicicola* which are responsible for significant yield losses. Till date, no resistant source is available against *Alternaria* blight, hence plant breeding methods can not be used to develop disease resistant varieties. Jasmonate mediated signalling pathway, which is known to play crucial role during defense response against necrotrophs, could be strengthened in *Brassica* plants to combat the disease. Since scanty information is available in *Brassica-Alternaria* pathosystems at molecular level therefore, in the present study efforts have been made to model jasmonic acid pathway in *Arabidopsis thaliana* to simulate the dynamic behaviour of molecular species in the model. Besides, the developed model was also analyzed topologically for investigation of the hubs node. COI1 is identified as one of the promising candidate genes in response to *Alternaria* and other linked components of plant defense mechanisms against the pathogens. The findings from present study are therefore informative for understanding the molecular basis of pathophysiology and rational management of *Alternaria* blight for securing food and nutritional security.

## Introduction

*Brassica species* include major group of oilseed crops being grown in 53 countries across the six continents in world, with India being the second largest grower after China^1,2^. Despite that, India has to import large amount of vegetable oils to meet the annual edible oil needs. In future, the demand for oilseeds production is likely to go up significantly due to increase in population^3,4^. One of the ways to increase productivity of mustard crops is to avoid the losses caused by various biotic and abiotic stresses^5,6^. Fungi and Oomycete are the main pathogens causing major yield losses in oil seed crops; more than thirty diseases are incurred in *Brassica* crops in India^7^. *Alternaria* blight, white rust, downy mildew and powdery mildew hold major importance on the basis of their wide distribution and yield losses^8,9^. Of these, *Alternaria* blight disease caused by *Alternaria brassicae* and *Alternaria brassicicola* is responsible for significant yield losses of the *Brassica* species all over the world. Depending upon severity, the yield losses have been reported to range from 35% to 46% in India and up to 70% all over the world with no proven source of transferable resistance in any of the hosts^10–12^. Disease management strategies utilizing fungicidal chemicals are practically insufficient in addition to being environmentally hazardous. Rapid evolution of the new pathogenic strains has further complicated breeding for resistance in crop plants. *Alternaria brassicae* is a necrotrophic pathogen which produces lesions on leaves, stem and siliquae affecting quantity as well as quality of seed by reducing oil content, size and colour^13^.

Plants respond locally to biotic stresses using inducible basal defense networks triggered through recognition and response to conserved pathogen-associated molecular patterns (PAMP) *via* Mitogen Activated Protein Kinases (MAPKs)^14–17^. In addition, immunity can be induced in tissues remote from the sites of infection by systemic acquired resistance, initiated after gene-for-gene recognition between plant resistance proteins and pathogen effectors^14^. Jasmonates and its functional analogs are known to play key roles for systemic defense, possibly acting as the initiating signal for systemic acquired resistance^14^. Jasmonic acid accumulates rapidly in phloem exudates of the leaves treated with an avirulent strain of *Pseudomonas syringae* in *Arabidopsis thaliana*, the transcripts associated with biosynthesis of jasmonate are up-regulated within 4 h, and increases transiently. The systemic defense can be mimicked by accumulation of JA during interaction of plant systems with unknown molecules; such activity was not shown in JA mutant plants.

Jasmonates play tremendous role in protecting plants from pathogen attack by receiving signals through MAPKs^18^. JA synthesis is initiated in the chloroplasts, while most MAPKs are cytosolic; thus, MAPKs are likely to transmit the extracellular elicitor signal for biosynthesis of JA in plants^18,19^. The biosynthesis of jasmonic acid takes place in three subcellular compartments: it is initiated in the chloroplast, followed by the peroxisome, and finally the cytoplasm. It starts with the 18C fatty acid α-linolenic acid 18:3 (α-LA). The first step involves release of the α-LA from galacto- and phospholipids situated at the chloroplast membrane by the enzymatic action of phospholipases (PLAs), which contain DEFECTIVE IN ANTHER DEHISCENCE 1 (DAD1) in *Arabidopsis thaliana* ^20^. After that, the polyunsaturated fatty acids α-LA is oxidized by 13-LIPOXYGENASE (LOX) leading to the formation of 13-hydroperoxy-9,11,15-octadecatrienoicacid (13-HPOT)^21–23^. Subsequently, 13-HPOT is converted into the stable cis(+)-oxophytodienoic acid (cis-OPDA) intermediate by two different enzyme families called as ALLENE OXYDE SYNTHASE (AOS) and ALLENE OXIDE CYCLASE (AOC)^24–27^. After that the cis(+)-oxophytodienoic acid (cis-OPDA) is transported from chloroplast to peroxisome. However, how cis-OPDA changes its subcellular compartment is largely unidentified. To date, only one gene, a peroxisome-localized protein of the ATP binding cassette (ABC) transporter class, termed as COMATOSE has been linked with subcellular transport of JA^28,29^. On the other hand, as loss of function mutants (in *Arabidopsis thaliana*) can still produce some JA, it suggests that most probably other transporters are also involved^30^. The cis-OPDA is reduced by an enzyme called OPDA REDUCTASE (OPR) and subsequently undergoes three rounds of β-oxidation by the action of ACYL-CoA OXIDASE (ACX) leading to the manufacture of jasmonic acid (JA)^31,32^. JA is then transported from peroxisome to cytoplasm *via* unknown route where it can be transformed by several enzymes^33^. The best example is GRETCHEN HAGEN 3s (GH3s), which conjugates JA with various amino acids mostly isoleucine, leading to the production of bioactive JA-Ile molecule^34–37^, which activate the expression of JA responsive genes.

Recent advances in omics science and technology have produced wealth of information about plant-pathogen interactions in the model plant *Arabidopsis thaliana* at molecular level, which may be utilized for deciphering the complexity of jasmonic acid signalling triggered during pathogenesis of *Alternaria* species of *Brassica* that enables us to identify possible molecular targets. These targets will further be exploited to develop strategies for induction of *de novo* defense in crop plants during pathogenesis^5,38^. It is a demand of time to harness the potential of systems biology for decoding the resistance machinery in *Brassica* through modeling of jasmonate signalling pathway in *Arabidopsis thaliana* for sustainable agriculture.

In view of above fact, a model of JA signalling pathway with respect to plant-pathogen interaction has been developed to predict the pathophysiology of *Alternaria* blight. The integrated pathways help to reduce the effects of external perturbation to whole plant systems due to its robust properties^39^. Each species in the pathway is considered as node and linked to other nodes at defined rate kinetics^40^. The aim of metabolic modelling of Jasmonate signaling pathway is to provide a hypothesis based on computational simulation at various time extents and network analysis of *Arabidopsis-Alternaria* interaction for identification of key regulatory element present in the pathway^15^. The integrated pathways will prove useful in understanding the pathophysiology of *Alternaria* blight disease in *Brassica* ^5^. It is important to determine the key properties and parameters which are used to predict the dynamics of model^40^. Compatibility of quantity and type of data utilized in the model is essential to meet the model parameters^41^. It is wonderfully established that the functional molecules of the living systems such as DNA, RNAs, receptor, enzymes, hormones, metabolites *etc*., are necessary for the integration of network to modulate behavior of the metabolic pathways^42^. Various *In silico* comprehensive maps^15,40,43–48^ have been constructed for dynamic analysis, pathophysiology and control mechanism as well as identification of biomarker for disease management. In the present study attempts were made to develop jasmonate mediated pathway model for functional characterization of components involved in induction of defense against *Alternaria* blight in *Brassica*.

## Results

### Description of constructed model

**Figure 1:**
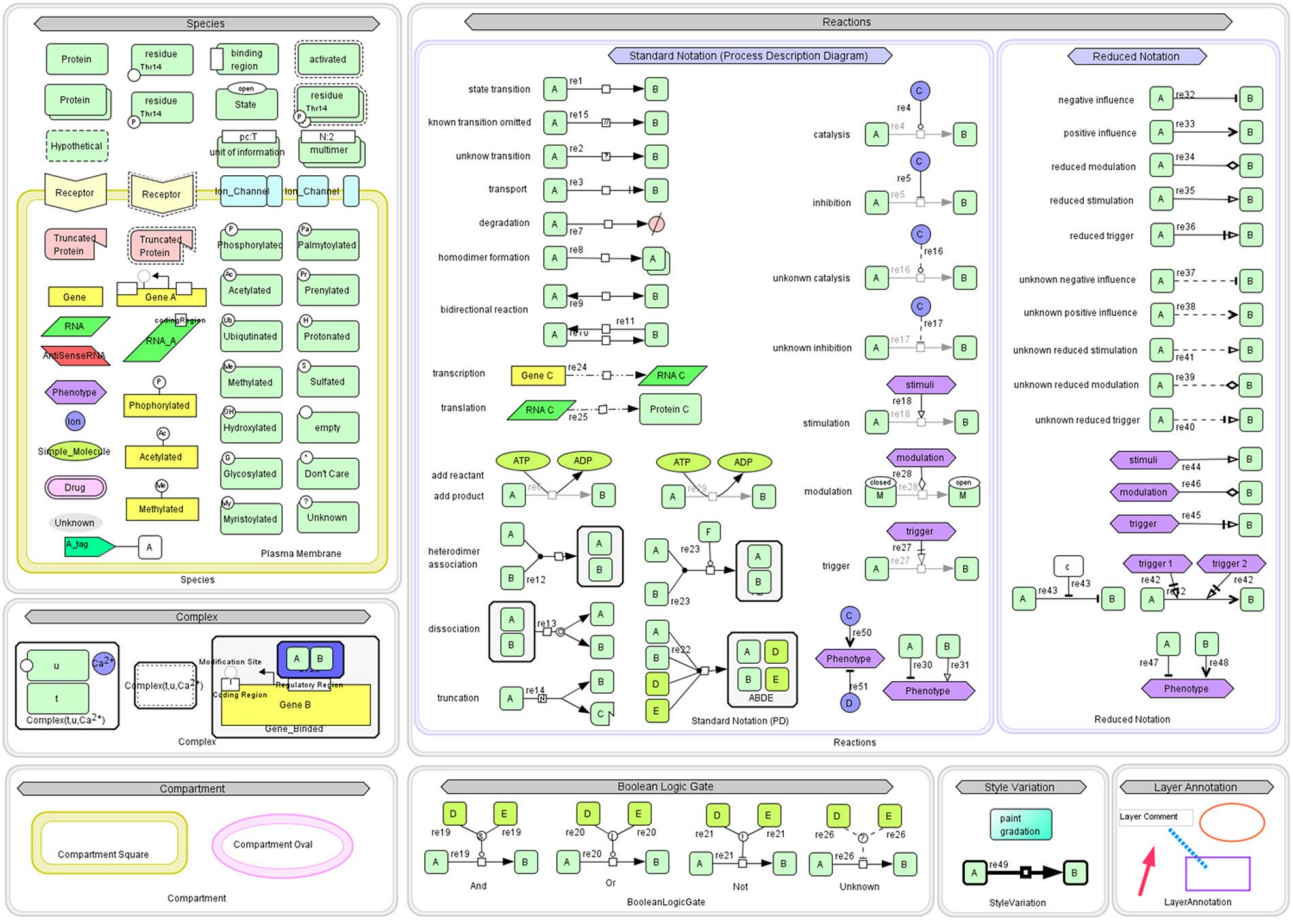
Systems Biology Graphical Notation (SBGN) symbols provided in CellDesigner4.4 for modeling of biological pathway.

**Figure 2:**
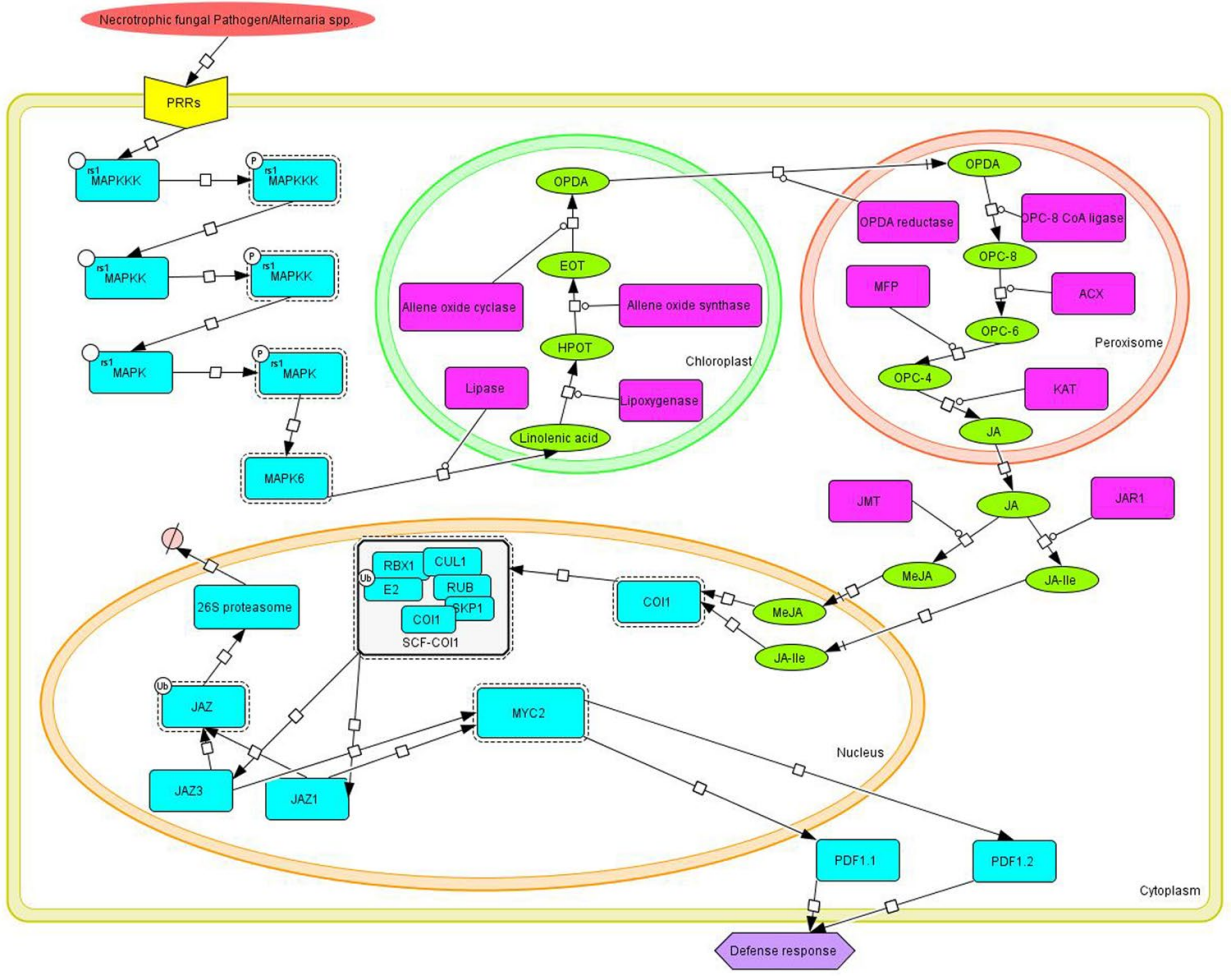
Jasmonate singaling pathway map was constructed by CellDesigner4.4 using systems biology graphical nonation (SBGN). A total number of 37 reactions and 44 species were included. The process diagrams, explicitly displaying unknown molecule, receptors, proteins, protein in phosphorylated forms, simple molecules and different cellular compartment. The active state of the molecules is indicated by a dashed line surrounding the molecule and defense response in the form of phenotype symbol with different colors and shape.

To date, a JA signaling model using Systems Biology Graphical Notation (SBGN) has not been constructed^49^. The goal of this study is to decipher the complexity of *Arabidopsis-Alternaria* pathosystem for understanding the pathophysiology of *Alternaria* blight in *Brassica* through quantitative and qualitative analysis of jasmonate biosynthesis pathway in terms of upstream signal and downstream response produced during pathogenesis process. These molecular mechanisms were modelled based on system biology approach to determine the duration of JA responses as well as try to identify key components involved in regulation of pathway during pathogenesis for achieving resistance against the disease. SBGN was utilized to assemble the relationship among different molecular species in the model based upon previous studies mined from scientific literatures and databases (Fig. 1). The model consists of 4 compartments, 44 species, 28 proteins, 14 simple molecules, 1 phenotype, and 37 reactions (Fig. 2).

### Dynamic behaviour prediction of constructed model through simulation analysis

Present study explains the outcome of integrated systems based approach that explored the intricate nature of jasmonate signalling pathway during pathogenesis at molecular level. The rate laws produced by SBMLsqueezer were utilized to predict the dynamic behavior of the pathway **(**Table 1 **)**. This could be useful for understanding the pathophysiology of *Alternaria* blight in *Brassica* for developing an efficient disease management strategy. The simulation jobs were run on hardware configuration comprises of intel corei3 processor of 2.40 GHz and 4GB RAM on 64 bit windows operating system laptop.

**Table 1:** Reactions and details of kinetics rate equations used in the model.

SN	Reactions	Kinetics equations
1	Necrotrophic fungal pathogen → PRRs	Vre1 = vmax_re1 × Necrotrophic fungal pathogen/(kmc_re1_ Necrotrophic fungal pathogen + Necrotrophic fungal pathogen)
2	PRRs → MAPKKK	Vre2 = vmax_re2 × PRRs/(kmc_re2_PRRs + PRRs)
3	MAPKKK → MAPKKK	Vre3 = vmax_re3 × MAPKKK/(kmc_re3_MAPKKK + MAPKKK)
4	MAPKKK → MAPKK	Vre4 = vmax_re4 × MAPKKK/(kmc_re4_MAPKKK + MAPKKK)
5	MAPKK → MAPKK	Vre5 = vmax_re5 × MAPKK/(kmc_re5_MAPKK + MAPKK)
6	MAPKK → MAPK	Vre6 = vmax_re6 × MAPKK/(kmc_re6_MAPK + MAPK)
7	MAPK → MAPK	Vre7 = vmax_re7 × MAPK/(kmc_re7_MAPK + MAPK)
8	MAPK → MAPK6	Vre8 = vmax_re8 × MAPK/(kmc_re8_MAPK + MAPK)
9	MAPK6 → Linolenic acid catalyzed by Lipase	Vre9 = Lipase × kcat_re9 × MAPK6/(kmc_re9_MAPK6_Lipase + MAPK6)
10	Linolenic acid → HPOT catalyzed by Lipoxygenase	Vre10 = Lipoxygenase × kcat_re10_s13 × Linolenic acid/(kmc_re10_Linolenic acid_Lipoxygenase + Linolenic acid)
11	HPOT → EOT catalyzed by Allene oxide synthase	Vre11 = Allene oxide synthase × kcat_re11_s15 × HPOT/(kmc_re11_HPOT_Allene oxide synthase + HPOT)
12	EOT → OPDA catalyzed by Allene oxide cyclase	Vre12 = Allene oxide cyclase × kcat_re12_s15 × EOT/(kmc_re12_EOT_Allene oxide cyclase + EOT)
13	OPDA → OPDA catalyzed by OPDA reductase	Vre13 = OPDA reductase × kcat_re13_s19 × OPDA/(kmc_re13_OPDA_OPDA reductase + OPDA)
14	OPDA → OPC-8 catalyzed by OPC-8 CoA ligase	Vre14 = OPC-8 CoA ligase × kcat_re14_s21 × OPDA/(kmc_re14_OPDA_OPC-8 CoA ligase + OPDA)
15	OPC-8 → OPC-6 catalyzed by ACX	Vre15 = ACX × kcat_re15_s23 × OPC-8/(kmc-re15_OPC-8_ACX + OPC-8)
16	OPC-6 → OPC-4 catalyzed by MFP	Vre16 = MFP × kcat_re16_s25 × OPC-6/(kmc_re16_OPC-6_MFP + OPC-6)
17	OPC-4 → JA catalyzed by KAT	Vre17 = KAT × kcat_re17_s27 × OPC-4/(kmc_re17_OPC-4_KAT + OPC-4)
18	JA → JA	Vre18 = vmax_re18 × JA/(kmc_re18_JA + JA)
19	JA → MeJA catalyzed by JMT	Vre19 = JMT × kcat_re19_s30 × JA/(kmc_re19_JA_JMT + JA)
20	JA → JA-IIe catalyzed by JAR1	Vre20 = JAR1 × kcat_re20_s32 × JA/(kmc_re20_JA_JAR1 + JA)
21	MeJA → MeJA	Vre21 = vmax_re21 × MeJA/(kmc_re21_MeJA + MeJA)
22	JA-Ile-JA-Ile	Vre22 = vmax_re22 × JA-Ile/(kmc_re22_JA-Ile + JA-Ile)
23	JA-Ile → COI1	Vre23 = vmax_re23 × JA-lle/(kmc_re23_JA-Ile + JA-Ile)
24	MeJA → COI1	Vre24 = vmax_re24 × MeJA/(kmc_re24_MeJA + MeJA)
25	COI1 → SCF-COI1	Vre25 = vmax_re25 × COI1/(kmc_re25_COI1 + COI1)
26	SCF-COI1 → JAZ3	Vre26 = vmax_re26 × SCF-COI1/(kmc_re26_SCF-COI1 + SCF-COI1)
27	SCF-COI1 → JAZ1	Vre27 = vmax_re27 × SCF-COI1/(kmc_re27_SCF-COI1 + SCF-COI1)
28	JAZ3 → JAZ	Vre28 = vmax_re28 × JAZ3/(kmc_re28_JAZ3 + JAZ3)
29	JAZ1 → JAZ	Vre29 = vmax_re29 × JAZ1/(kmc_re29_JAZ1 + JAZ1)
30	JAZ → 26 S proteasome	Vre30 = vmax_re30 × JAZ/(kmc_re30_JAZ + JAZ)
31	26 S proteasome → Degrade	Vre31 = vmax_31 × [26 S proteasome] × vol(Nucleus)/(kmc_re31_[26 S proteasome] + [26 S proteasome] × vol(Nucleus))
32	JAZ1 → MYC2	Vre32 = vmax_re32 × JAZ1/(kmc_re32_JAZ1 + JAZ1)
33	JAZ → MYC2	Vre33 = vmax_re33 × JAZ/(kmc_re33_JAZ + JAZ)
34	MYC2 → PDF1.2	Vre34 = vmax_re34 × MYC2/(kmc_re34_MYC2 + MYC2)
35	MYC2 → PDF1.2	Vre35 = vmax_re35 × MYC2/(kmc_re35_MYC2 + MYC2)
36	PDF1.1 → Defense response	Vre36 = vmax_re36 × PDF1.2/(kmc_re36_PDF1.1 + PDF1.1)
37	PDF1.2 → Defense response	Vre37 = vmax_re37 × PDF1.2/(kmc_re37_PDF1.2 + PDF1.2)

The real values for each molecular species in the model have not been used due to un-availability of experimental data for an individual cell. Simulation can decipher the behaviour of key molecular species in the presence and absence of pathogen elicitor for a particular disease. Defense responses through jasmonate signalling were predicted during pathogenesis of *Alternaria* blight in *Arabidopsis thaliana*.

In the absence of quantitative molecular data of an individual cell, it is difficult to estimate the relationship between species. Therefore, quantity is occupied in terms of amount to confirm their existence^40^. The values for each molecular species in the model ranged from 0.25 to 2.5. The value of pathogen is set at 0.25; receptor is set to equal of 0.5 due to its basal amount. The amounts of species are set at 0.5, 1 and 2, according to their activation and inactivation states, for example non-phosphorylated MAPKs are set at 0.5 and phosphorylated are set at 1; all the metabolites are set at 0.5 and all the enzymes involved in biosynthetic pathway are set at 1. The value of JA, MeJA and JA-IIe are considered as variable species and its values range from 0.5 to 1 to observe the dynamic behaviour of system. The value of COI1 is set in the range from 0.0 to 2 and that of SCF-COI1 complex is equal to 1.5; the amount of transcription factors is set to 1 and proteins is set to 2 as well as the amount of phenotypic response is set equal to 2.5.

**Figure 3:**
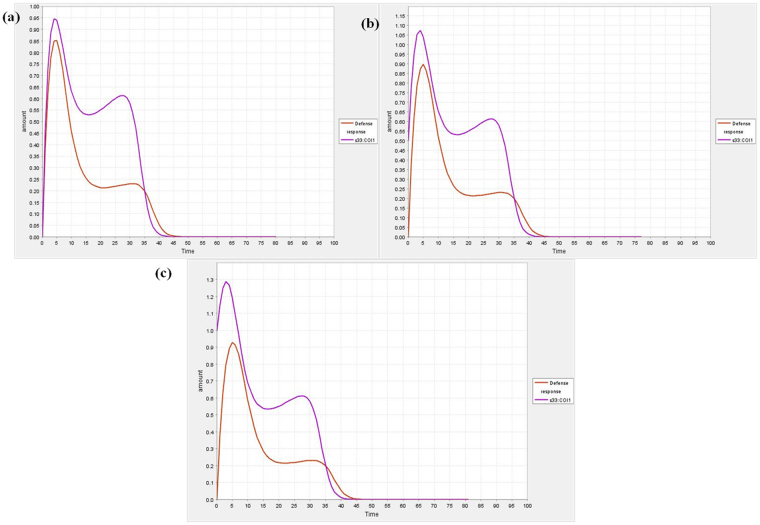
Dynamic behaviour analysis of defense response (**a**) COI1 is set at 0.0 (**b**) COI1 is set at 0.5 (**c**) COI1 is set at 1.0.

**Figure 4:**
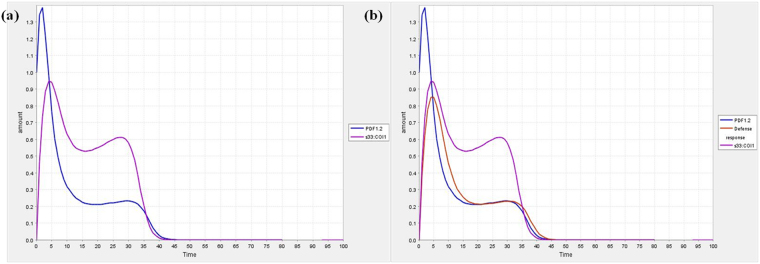
Simulation curve for (**a**) PDF1.2 with respect to COI1 (**b**) PDF1.2 and defense response with respect to COI1; the amount of PDF1.2 is set at 1.0.

Previous studies clearly demonstrated that when the intensity of abiotic and biotic stresses increases, the defense responses decrease rapidly in time dependent manner^15^. Here, the dynamic behaviour of the different species in the model was visualized during simulation with course of time. The variation in defense response with respect to COI1 is revealed in Fig. 3. When the COI1 interacts with jasmonate signal, it controls the modulation behaviour of various gene(s) involved in defense responses. It was predicted that when the expression level of COI1 increases the defense responses also increases, besides, when the expression of COI1 is slowed down the regulation of defense responses is down regulated in time dependent manner during plant-pathogen interaction (Fig. 3). The dynamic behaviour of plant system with respect to pathogenesis of *Alternaria* species and its effect on expression level of COI1 was shown in Fig. 3a,b, and c. PDF1.2 is one of plant defense gene playing essential role in disease resistance, during simulation analysis, it was predicted that its activity is also controlled by the activation of COI1. It was also predicted that when the expression of COI1 increases, the PDF1.2 is up- regulated. The fluctuations in the dynamic behaviour of PDF1.2 with respect to COI1 were also observed in different time scale (Fig. 4a and b).

**Figure 5:**
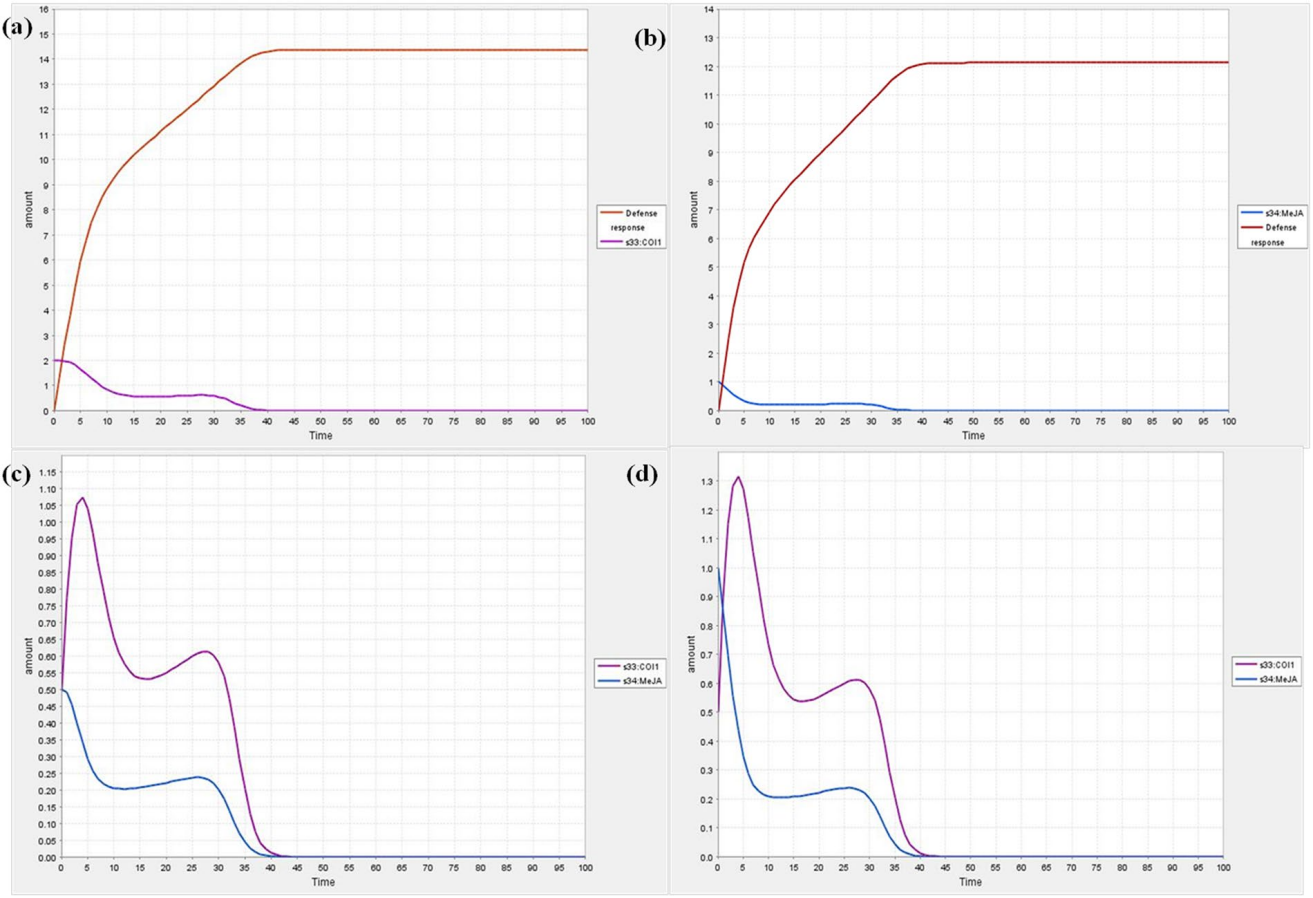
Dynamic behaviour of defense response with respect to COI1 and COI1 dynamics with respect to Me-JA (**a**) COI1 amount is set at 2.0 (**b**) Me-JA amount is set at 1.0 (**c**) Me-JA amount is set at 0.5 (**d**) Me-JA amount is set at 1.0.

The model has also perturbed with the variable quantity of COI1 to confirm its role in the regulation of defense responses quantitatively. It was found that the higher expression of COI1 modulates the cellular expression of defense related genes and provides protection to the crop plant systems (Fig. 4). It is well characterized that the COI1 is jasmonate receptor and responsible for triggering jasmonic acid mediated immunity in *Arabidopsis thaliana* through interaction with jasmonate signal and its expression in balanced way is essential to crop plants for defending pest and pathogen attack^6^. Present study decoded the fluctuation of COI1 at different amount with respect to time that can useful to maintaining its expression level in the balanced way during pathogenesis in future. Jasmonic acid and its structural analogs are known natural substrates that initiates the activity of COI1; Me-JA is one of the functional analogs of jasmonic acid which is known to promote COI1 interaction with JAZ1, which is responsible for defense responses, thus, we perturbed the model with different amount of Me-JA to predict the dynamic behaviour of COI1 (Fig. 5a). It was found that the dynamic activity of COI1 is controlled by the production of jasmonic acid and activity of its functional analogs (Fig. 5b,c and d).

### Topological analysis of JA signalling network

**Figure 6:**
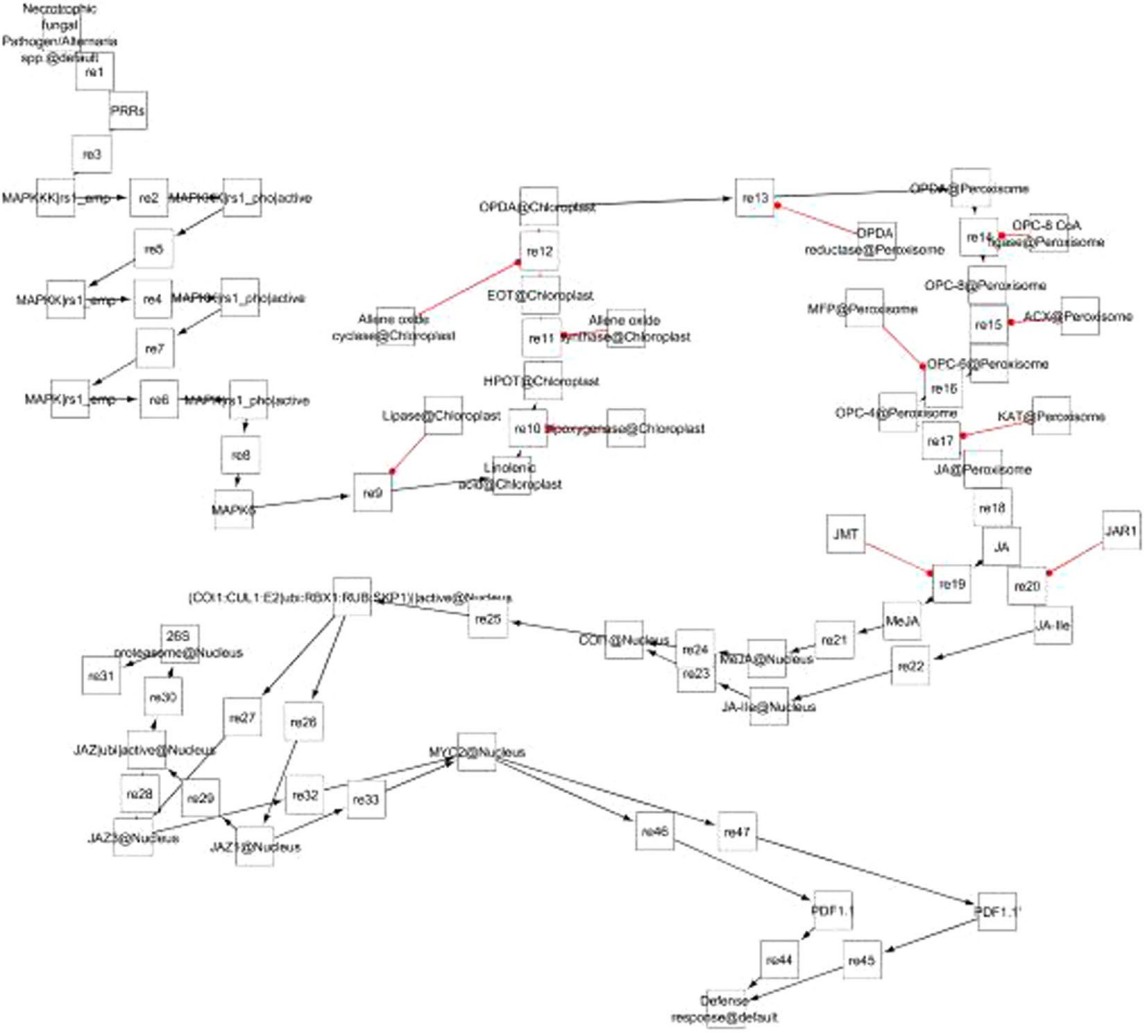
Module style view of JA signalling network (directed graph) node (square box) with catalysis (red color), and physical stimulation (black color).

The molecular interaction network of jasmonic acid has 81 nodes and 84 edges and all of the data were taken as scale free properties as anticipated from biological network^50,51^ (Fig. 6). The average path length was used to measure the JA signalling network as directed and un-directed graph. The in- and out degree distributions for modelled network have power low exponent with approximate value a = 54.000, b = −1.848 and a = 74.000, b = −3.888 (R-squared value 1.000 and correlation 1.0) respectively. R-squared was computed on logarithmized value. Out and in degree power low distribution of JA network was analyzed, which explains the number of contacts per node, in order to determined hubs and verify the scale-free property^52,53^.

**Figure 7:**
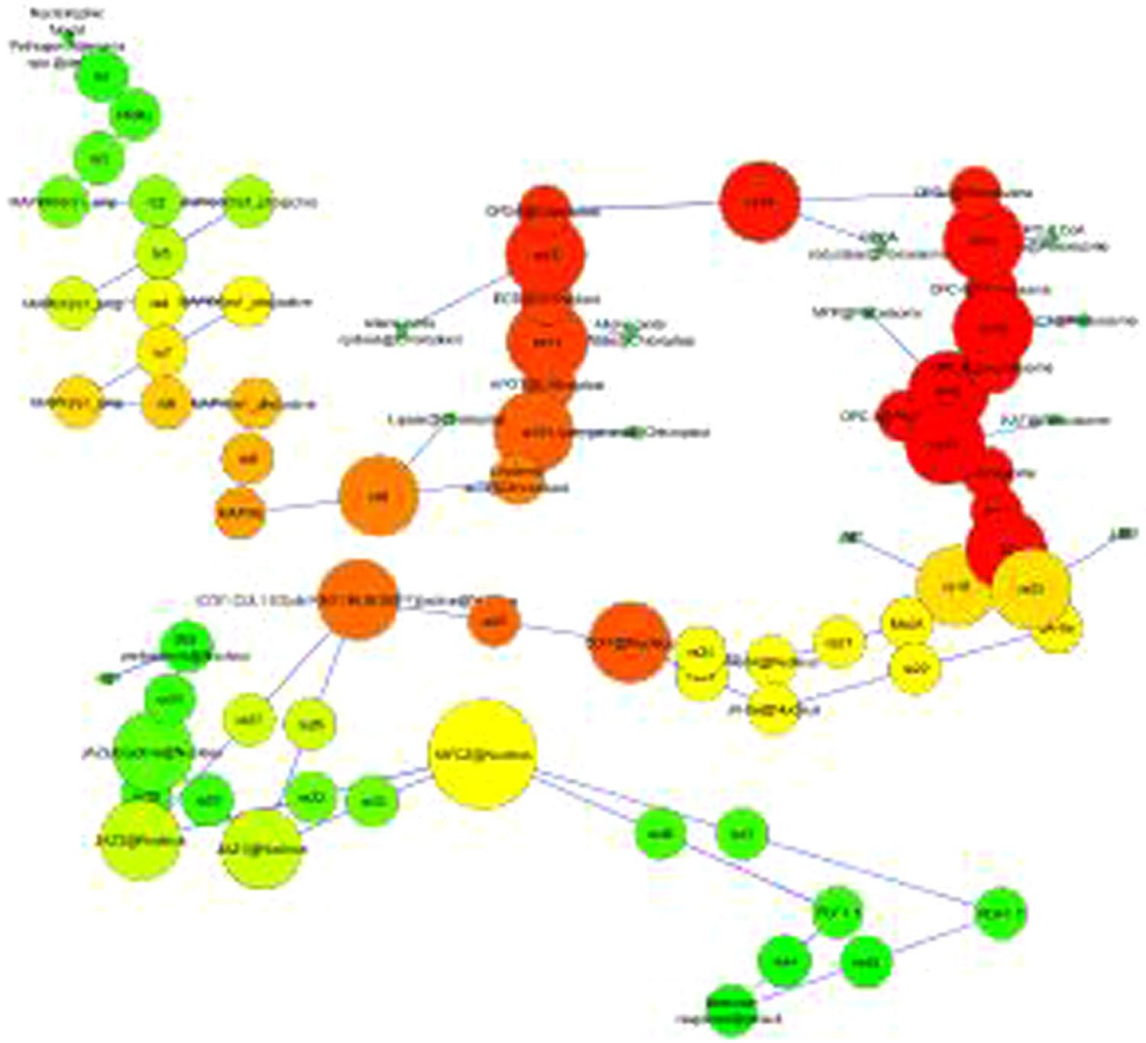
Mapping of node in JA signaling network: Out degree visualization of whole pathway to map hub nodes.

Network analysis revealed that the JA network has 81 node, 84 edges, 1 connected component node, 0 isolated node, 2.074 average number of neighbours, 6480 shortest path, 17.822 characteristics path length and 52 network diameter (Table 2). The visualize parameter of NetworkAnalyzer was used to map the hub node in the network using the visual style to map node size “Degree” and node color “BetweenessCentrality” to determined the hub nodes. The BetweennessCentrality of each node is defined as a number between 0 and 1. It reveals the amount of control that this node exerts over the interactions of other nodes in the network^54^. The node JAZ1, JAZ3 and MYC2 was found as a hub node, which might play, a key regulatory role in Jasmonic acid mediated immunity (Fig. 7). Modeling and analysis of these networks would decode the complexity of agricultural trait and help us to bring out the hidden properties of the crop plant systems. A resolution image of whole network in modular view along with predicted hub nodes is shown in Fig. 7.

**Table 2:** Values of topological parameters for JA signalling networks.

**Node**	84	**CPL**	17.822
**Edge**	84	**ND**	52
**CC**	1	**MENP**	0
**ANN**	2.074	**IN**	0
**SP**	2768	**NR**	26

CC, connected component; ANN, average number of neighbors; SP, shortest path; CPL, characteristics path length; ND, network diameter; MENP, multi-edge node pair; IN, isolated node; NR, network radius.

## Discussion

*Alternaria* blight is one of the major threats to attain productivity of crucifers crops mainly Oilseed *Brassica* species such as Indian mustard, cabbage, broccoli, and oilseed rape. The yield losses from this disease could reach up to 80%^55^. Till date disease management through fungicide chemicals was the only option but these are practically insufficient in addition to being environmentally hazardous. In many European countries, organic agriculture has been transformed quickly through agricultural policy, which reports indicate opportunity of biological management of the disease^9^. Certain wild type plants in *Brassica* and *Arabidopsis* are known to show resistance or incompatible reactions to *Alternaria*. But their transferability to the agronomically important crops has been a limiting factor through breeding strategies due to several reasons including, genetic drag and self incompatibility, arising a major problem all over the world with no proven source of transferable resistance in any of the hosts^56,57^.

It is being felt that *Alternaria brasscicola* - *Arabidopsis* could be used as one of the excellent model system to understand phathophysiology of Alternaria blight in *Brassica*. *Alternaria brassicae*, being a hemibiotroph fungus displays necrotrophy at some stages of pathogenesis. It has been reported that Jasmonic acid plays important role in mounting the defense response towards necrotrophic fungal pathogens^17^. Deep insight in to the JA mediated signal transduction pathway involved during defense response will help in devising strategies for development of resistance against Alternaria blight provided that the key molecular components have been identified. The higher expression of defense related genes during pathogenesis process caused by *A. brassicae* would have an immense potential to impart fungal resistance in the crop plants through their over expression^58^.

Recent advances in integrated sciences have huge potential to handle such recalcitrant problem; therefore, the important components triggered during disease were investigated in the present study *via* modeling and simulation as well as network analysis of jasmonate signaling pathway in *Arabidopsis thaliana*. Jasmonate signaling pathway is well characterized pathway in plant systems for defense responses particularly in case of necrotrophic pathogens but the knowledge about the key element of pathway during pathogenesis in term of quantity remain unclear^5^. The developed model clearly demonstrated the fluctuations of key component involved in diseases resistance during simulation besides network analysis were also conducted to determine the value of topological parameter of the JA network for mapping of hubs based on out degree visualization^59^. Degree is defined as a number of edges linked to a node, if it is a self loop; it shall be counted as two edges of a single node. In directed node, the degree distribution is of two types, in and out degree distribution. The number of coming edges to a node is called in-degree distribution and number of out-going edges is called out degree distribution of a node^60^. In case of biological network, the node and edge is defined in different ways; in metabolic network, the node is considered as metabolites and reactions are considered as edges; in protein-protein interaction network, protein is considered as node and edges are considered as bonding between one protein with other protein. Whereas, in case of gene regulatory network, the nodes are considered as gene and edges are known for its regulatory effect^52^.

The aim of modeling and simulation is to describe and understand the pathophysiology at molecular level where key regulatory element and its complex mechanisms that happen at several time scales were predicted. During simulation analysis at variable amount of jasmonic acid and its functional analogs, Me-JA; COI1 is predicted as one of the potential molecular target because it controls the expression of various defense related genes during pathogenesis. It was also predicted that the up and down regulation of COI1 during plant-pathogen interaction is totally linked to amount of jasmonic acid and its functional analogs. Besides, we have manually increased and decreased the amount of COI1 to determine the behaviour of system during pathogenesis^61^. Recent cloning and expression study conducted on *COI1 gene* of *Aquilaria sinensis* demonstrated that the Methyl jasmonate (MeJA), mechanical wounding and heat stress could significantly induce its expression level^62^. Network analysis has been publicized to be one of the powerful tools for decoding the key regulatory elements (hubs), which is responsible for controlling the complex biological machinery during diseases^63^. Hub is a concept originated from the field of network science that refers to a node having number of links. Identification of hub nodes in complex networks has attracted a growing attention over the last decade^64,65^. In biology, a hub is metabolite, gene, or protein which is highly connected to the other nodes (is metabolite, gene, or protein) in a network and that is known to regulates several other pathways associated with the main pathway. Recent advances in plant disease resistance research have provided exciting novel insights into decoding of defense signaling network. Diverse number of components plays essential roles in the regulation of whole network through cross-communication in antagonistic or synergistic manners. Biological properties of hubs in JA signalling pathway are very significant as these are closely related to the well characterized components linked to the defense responses. Our finding demonstrated the highly connected nodes (hubs) such as JAZ1, JAZ3 and MYC2 but it was predicted that the expression of these hubs is managed by COI1. These hub nodes are involved in the regulation of defense machinery through interaction of COI1 with jasmonate signal. For that reason, COI1 is considered as one of the key components of JA pathway. Previous study suggested that COI1 has the structural traits for binding to jasmonyl-isoleucine (JA-IIe/Coronatine)^66^ and determined as an intracellular signal with a hormone binding site^67^. Therefore, it may serve as a potential molecular target for triggering *de novo* resistance in crop plant systems during plant-pathogen interaction *via* engineering of jasmonic acid pathway for sustainable production of jasmonic acid and its functional analogs during pathogenesis or providing exogenous compounds as defense induces that can mimick the expression of COI1.

It is evident that small molecule targeting COI1 leads to the production of appropriate defense whereas engineering of jasmonic acid pathway for proper production of jasmonic acid during diseases has a very complex and time taking process, therefore, we have concluded that there is a need of novel defense inducer molecule more efficient and stable as compared to jasmonic acid and its structural analogs to tackle today’s problem of pathogen resistance by developing *de novo* resistance in crop plants for robust agricultural productivity and sustainability^5,6^. Biologically active small molecules have been verified previously for crop plant protection by producing *de novo* resistance^68,69^. In our knowledge, we have modeled JA signalling pathway first time to analyze the whole systems during plant-pathogen interaction. This integrated approach shows that computational demonstration can construct and explain precisely the complete observable fact of JA signalling during pathogenesis at molecular level. The constructed technical model describes the biochemical relationship of different molecular species and effect of pathogen in different unit time. These types of *In silico* finding provides a valuable hypothesis and mimics by the simulation analysis as *in vitro* test. The study exhibits that cellular reaction depends on the effective quantity of jasmonic acid and its functional analogs inside the cell^70^. As a result, the understanding of the integrated behaviour of model can be valuable to develop novel plant health and disease management strategies to fulfilling the demand of *Brassica* oilseed for rapidly growing world population.

COI1 was found as the most important molecular target. It can be utilizing to mimick jasmonic acid mediated immunity through engineering of the plant system or designing of the cost effective molecule, more efficient than naturally produced jasmonic acid for triggering *de novo* resistance during pathogenesis. During analysis, COI1 was found to control the expression of many defense related component in the model. Besides, other identified hubs node may also utilized for further investigation with respect to plant-pathogen interaction. A number of undiscovered effects of JA with their perturbed amount to its respective target can be predicted. This finding can be useful for experimental design and interpretation by using diseased and healthy plants. Nevertheless, it seems to be essential to examine more conditions that imitate realistic stress situation in an attempt to find out which methods need to be developed to cope with their usual environment. In term of agricultural significance, it is assumed that the availability of a constructed model further speed up the research for crop protection. Such type of models can be utilize in identification of molecular target and its dynamics at cellular and system level for management of *Alternaria* blight: a recalcitrant disease of *Brassica* through designing of defense inducer molecules that can able to trigger defense related pathway or application of genetic engineering approaches for sustainable agriculture.

## Methods

### Model construction

A comprehensive literatures survey was done to determine the relationship between different molecular components involved in Jasmonic acid biosynthesis pathway that triggers defense responses during plant-pathogen interaction in *Arabidospis thaliana*. A Systems Biology Graphical Notation (SBGN) for building of biological networks to express adequate information in a clearly visible and precise way was used^49^ (Fig. 1). The Metabolic map of Jasmonic acid signalling pathway was constructed by CellDesigner4.4 and stored in Systems Biology Markup Language (SBML), a machine-readable format for representation of the biological network^71–75^. CellDesigner is a process diagram editor tool for representation of biological pathways using inbuilt symbols of systems biology graphical notation (Fig. 1). It is a graphical user interface that facilitates systems biologist to utilize available symbols of DNA, RNA, Protein, Simple molecule, Catalysis, Stimulation, Inhibition, Phosphorylation, activation, degradation *etc* for construction of pathway. The control panel menu in CellDesigner provides us simulation facilities by using SBML ODE Solver and Copasi, It facilitates to specify the particulars of constraints, varying quantity and leading parameter search as well as shared simulation with instinctive way^40^.

### Assignment of kinetic rate equations

SBMLsqueezer_v2.1 was applied to generate kinetic rate equations for each reaction of the constructed model through CellDesigner. This approach assists the modeling steps *via* programmed generation of equation and takes over the highly error-prone and complicate process of manually assigning kinetic equations^76^. It is a CellDesigner plugin that uses information from the system biology graphical notation (SBGN) representation of all components of network. SBMLsqueezer judges the Systems Biology Ontology (SBO) annotations to pull out this information^40,77^. The rate laws that can be produced by SBMLsqueezer consist of numerous types of generalized mass action, and comprehensive as well as generalized enzyme kinetics **(**Table 1 **)**. Kinetic rate equations for metabolic reactions include uni–uni Michaelis–Menten kinetics, generalized hill equation for uni–uni reactions, Irreversible non-modulated non-interacting reactant enzymes, bi-uni enzyme reactions, bi–bi enzyme reactions, thermodynamics and convenience kinetics modular rate laws for enzymatic reactions^76,78^.

### Model simulation

To simulate the dynamic behavior of constructed model, SBML ODE Solver Library (SOSlib) was used through CellDesigner^71,79^, which enables us to run ordinary differential equations (ODE) based simulations. This is a frequently used method for quantitative analysis of biological systems in term of computational efficiency. SOSlib is a programming library for symbolic and numerical analysis of biochemical reaction network models encoded in the SBML^79^. CellDesigner is an integrated modeling and simulation platform that provides several third-party tools for interactive model simulation that is SOSlib^79^, COPASI^80^ and Simulation Core Library^81^. We have employed deterministic algorithm for our constructed model. In studying model of biological systems, we need solution for a given set of parameter values with their assessment for dependency of other species in the model. The simulation engine itself was executed by the native library, and the results of simulation analysis were shown in a Graphical User Interface (GUI) window written in JAVA programming language^75,82^.

### Network analysis

Jasmonate signaling model generated in CellDesigner was exported in SBML format, which was imported in the Cytoscape 2.8.3 using Biological Network Manager (BiNoM)^83^. BiNoM is a Cytoscape plug in^84^ to support the operation on biological networks represented in standard systems biology formats (SBML, SBGN, BioPAX) and to carry out studies on the complex network Structure^85^. Cytoscape is an open source software platform for integration, analysis and visualization of biological network^86^. Different types of plugins are available for various kinds of analysis, which facilitate us for decoding the complexity of biological networks. NetworkAnalyzer plugin^87^ was applied for investigating and visualizing the important components of JA signaling pathway triggered during plant-pathogen interaction for devising the strategies to develop effective management system against *Alternaria* blight of *Brassica* ^88^.

## References

[1] Shekhawat, K., Rathore, S. S., Premi, O. P., Kandpal, B. K. & Chauhan, J. S. Advances in agronomic management of Indian mustard (Brassica juncea (L.) Czernj. Cosson): an overview. *International Journal of Agronomy* (2012).

[2] Joshi, M. Textbook of field crops. PHI Learning Pvt. Ltd (2015).

[3] Boomiraj K, Chakrabarti B, Aggarwal PK, Choudhary R, Chander S (2010). Assessing the vulnerability of Indian mustard to climate change. Agriculture, ecosystems & environment.

[4] Kannusamy J, Abdulraheem A (2013). Production and import of edible vegetable oils in India: An assessment. EXCEL International Journal of Multidisciplinary Management Studies.

[5] Kumar A, Pathak RK, Gupta SM, Gaur VS, Pandey D (2015). Systems biology for smart crops and agricultural innovation: filling the gaps between genotype and phenotype for complex traits linked with robust agricultural productivity and sustainability. OMICS.

[6] Pathak RK (2017). *In Silico* identification of mimicking molecules as defense inducers triggering jasmonic acid mediated immunity against *Alternaria* blight disease in *Brassica* species. Frontiers in Plant Science.

[7] Saharan, G. S., Mehta, N., Meena, P. D., & Dayal, P. Alternaria diseases of crucifers: biology, ecology and disease management. Springer Science & Business Media, Singapore (2016).

[8] Saharan, G. S., Mehta, N., & Sangwan, M. S. (Eds). Diseases of oilseed crops. *Indus Publishing* (2005).

[9] Meena PD, Awasthi RP, Chattopadhyay C, Kolte SJ, Kumar A (2010). Alternaria blight: a chronic disease in rapeseed-mustard. Journal of Oilseed Brassica.

[10] Mishra A, Pandey D, Goel A, Kumar A (2010). Molecular cloning and in silico analysis of functional homologues of hypersensitive response gene (s) induced during pathogenesis of Alternaria blight in two genotypes of Brassica. J Proteomics Bioinform.

[11] Mishra A (2015). Expression analysis of MAP K 4 and MAP K 6 during pathogenesis of Alternaria blight in susceptible and tolerant genotypes of *Brassica juncea*. European Journal of Plant Pathology.

[12] Chandrashekar N, Ali S, Rawat S, Grover A (2015). Gene expression profiling of Arabidopsis thaliana chitinase genes in response to Alternaria brassicae challenge. Indian Phytopathology.

[13] Mathpal P, Punetha H, Tewari AK, Agrawal S (2011). Biochemical defense mechanism in rapeseed-mustard genotypes against *Alternaria* blight disease. J Oilseed Brass.

[14] Truman W, Bennett MH, Kubigsteltig I, Turnbull C, Grant M (2007). Arabidopsis systemic immunity uses conserved defense signaling pathways and is mediated by jasmonates. Proceedings of the national academy of sciences.

[15] Pathak RK, Taj G, Pandey D, Arora S, Kumar A (2013). Modeling of the MAPK machinery activation in response to various abiotic and biotic stresses in plants by a system biology approach. Bioinformation.

[16] Meng X, Zhang S (2013). MAPK cascades in plant disease resistance signaling. Annual Review of Phytopathology.

[17] Pandey D, Rajendran SRCK, Gaur M, Sajeesh PK, Kumar A (2016). Plant defense signaling and responses against necrotrophic fungal pathogens. Journal of Plant Growth Regulation.

[18] Hu X, Wansha L, Chen Q, Yang Y (2009). Early signals transduction linking the synthesis of jasmonic acid in plant. Plant signaling & behavior.

[19] Kandoth PK (2007). Tomato MAPKs LeMPK1, LeMPK2, and LeMPK3 function in the systemin-mediated defense response against herbivorous insects. Proceedings of the National Academy of Sciences.

[20] Ishiguro S, Kawai-Oda A, Ueda J, Nishida I, Okada K (2001). The defective in anther dehiscence1 gene encodes a novel phospholipase A1 catalyzing the initial step of jasmonic acid biosynthesis, which synchronizes pollen maturation, anther dehiscence, and flower opening in *Arabidopsis*. The Plant Cell.

[21] Caldelari D, Wang G, Farmer EE, Dong X (2011). Arabidopsis lox3 lox4 double mutants are male sterile and defective in global proliferative arrest. Plant molecular biology.

[22] Chauvin A, Caldelari D, Wolfender JL, Farmer EE (2013). Four 13‐lipoxygenases contribute to rapid jasmonate synthesis in wounded Arabidopsis thaliana leaves: a role for lipoxygenase 6 in responses to long‐distance wound signals. New Phytologist.

[23] Chauvin A, Lenglet A, Wolfender JL, Farmer EE (2016). Paired hierarchical organization of 13-lipoxygenases in Arabidopsis. Plants.

[24] Laudert D, Pfannschmidt U, Lottspeich F, Holländer-Czytko H, Weiler EW (1996). Cloning, molecular and functional characterization of Arabidopsis thaliana allene oxide synthase (CYP 74), the first enzyme of the octadecanoid pathway to jasmonates. Plant molecular biology.

[25] Lee DS, Nioche P, Hamberg M, Raman CS (2008). Structural insights into the evolutionary paths of oxylipin biosynthetic enzymes. Nature.

[26] Stenzel I (2012). ALLENE OXIDE CYCLASE (AOC) gene family members of Arabidopsis thaliana: tissue-and organ-specific promoter activities and *in vivo* heteromerization. Journal of experimental botany.

[27] Otto M, Naumann C, Brandt W, Wasternack C, Hause B (2016). Activity regulation by heteromerization of *Arabidopsis* allene oxide cyclase family members. Plants.

[28] Theodoulou FL (2005). Jasmonic acid levels are reduced in COMATOSE ATP-binding cassette transporter mutants. Implications for transport of jasmonate precursors into peroxisomes. Plant Physiology.

[29] Dave A (2011). 12-Oxo-phytodienoic acid accumulation during seed development represses seed germination in *Arabidopsis*. The Plant Cell.

[30] Larrieu A, Vernoux TQ&A (2016). How does jasmonate signaling enable plants to adapt and survive?. BMC biology.

[31] Castillo MC, Martínez C, Buchala A, Métraux JP, León J (2004). Gene-specific involvement of β-oxidation in wound-activated responses in *Arabidopsis*. Plant Physiology.

[32] Breithaupt C (2006). Crystal structure of 12-oxophytodienoate reductase 3 from tomato: self-inhibition by dimerization. Proceedings of the National Academy of Sciences.

[33] Wasternack C, Strnad M (2016). Jasmonate signaling in plant stress responses and development–active and inactive compounds. New Biotechnology.

[34] Staswick PE, Tiryaki I (2004). The oxylipin signal jasmonic acid is activated by an enzyme that conjugates it to isoleucine in *Arabidopsis*. The Plant Cell.

[35] Fonseca S (2009). (+)-7-iso-Jasmonoyl-L-isoleucine is the endogenous bioactive jasmonate. Nature chemical biology.

[36] Han, G. Z. Evolution of jasmonate biosynthesis and signaling mechanisms. *Journal of Experimental Botany* erw470 (2016). 10.1093/jxb/erw470 28007954

[37] Larrieu A, Vernoux T (2016). Q&A: How does jasmonate signaling enable plants to adapt and survive?. BMC biology.

[38] Pathak RK (2016). Molecular modeling and docking studies of phytoalexin (s) with pathogenic protein (s) as molecular targets for designing the derivatives with anti-fungal action on *Alternaria spp*. of *Brassica*. Plant Omics.

[39] Lehár J, Krueger A, Zimmermann G, Borisy A (2008). High‐order combination effects and biological robustness. Molecular Systems Biology.

[40] Gupta MK, Misra K (2013). Modeling and simulation analysis of propyl-thiouracil (PTU), an anti-thyroid drug on thyroid peroxidase (TPO), thyroid stimulating hormone receptor (TSHR), and sodium iodide (NIS) symporter based on systems biology approach. Network Modeling Analysis in Health Informatics and Bioinformatics.

[41] Ideker T, Galitski T, Hood L (2001). A new approach to decoding life: systems biology. Annual review of genomics and human genetics.

[42] Autiero I, Costantini S, Colonna G (2009). Modeling of the bacterial mechanism of methicillin-resistance by a systems biology approach. PLoS One.

[43] Oda, K., Matsuoka, Y., Funahashi, A. & Kitano, H. A comprehensive pathway map of epidermal growth factor receptor signaling. *Molecular systems biology* 1 (2005). 10.1038/msb4100014 PMC1681468 16729045

[44] Oda, K. & Kitano, H. A comprehensive map of the toll‐like receptor signaling network. *Molecular systems biology* 2 (2006). 10.1038/msb4100057 PMC1681489 16738560

[45] Calzone L, Gelay A, Zinovyev A, Radvanyi F, Barillot E (2008). A comprehensive modular map of molecular interactions in RB/E2F pathway. Molecular systems biology.

[46] Kaizu K (2010). A comprehensive molecular interaction map of the budding yeast cell cycle. Molecular systems biology.

[47] Caron E (2010). A comprehensive map of the mTOR signaling network. Molecular systems biology.

[48] Wu G, Zhu L, Dent JE, Nardini C (2010). A comprehensive molecular interaction map for rheumatoid arthritis. PLoS One.

[49] Kitano H (2003). A graphical notation for biochemical networks. Biosilico.

[50] Newman, M., Barabási, A. L. & Watts, D. J. The structure and dynamics of networks *Princeton University Press* (2006).

[51] Barabási AL (2009). Scale-free networks: a decade and beyond. Science.

[52] Barabasi AL, Oltvai ZN (2004). Network biology: understanding the cell’s functional organization. Nature reviews genetics.

[53] Vallabhajosyula RR, Chakravarti D, Lutfeali S, Ray A, Raval A (2009). Identifying hubs in protein interaction networks. PloS one.

[54] Yoon J, Blumer A, Lee K (2006). An algorithm for modularity analysis of directed and weighted biological networks based on edge-betweenness centrality. Bioinformatics.

[55] Nowicki M, Nowakowska M, Niezgoda A, Kozik E (2012). Alternaria black spot of crucifers: symptoms, importance of disease, and perspectives of resistance breeding. Vegetable Crops Research Bulletin.

[56] Kolte SJ, Singh MP, Awasthi RP (1999). Induction of resistance in mustard (Brassica juncea) against Alternaria black spot with an avirulent Alternaria brassicae isolate-D. European Journal of Plant Pathology.

[57] Mukherjee AK, Lev S, Gepstein S, Horwitz BA (2009). A compatible interaction of Alternaria brassicicola with Arabidopsis thaliana ecotype DiG: evidence for a specific transcriptional signature. BMC plant biology.

[58] Chandrashekar, N. Isolation and molecular characterization of PR gene pathogen-inducible promoter from Arabidopsis thaliana in response to Alternari (Doctoral dissertation, division of molecular biology and biotechnology national research centre on plant biotechnology indian agricultural research institute new delhi). Available at http://krishikosh.egranth.ac.in/handle/1/5810009893 (2014).

[59] Vehlow C (2015). Visual analysis of biological data-knowledge networks. BMC bioinformatics.

[60] Kohl, M., Wiese, S. & Warscheid, B. Cytoscape: software for visualization and analysis of biological networks. *Data mining in proteomics: from standards to applications* 291–303 (2011). 10.1007/978-1-60761-987-1_18 21063955

[61] Dada JO, Mendes P (2011). Multi-scale modelling and simulation in systems biology. Integrative Biology.

[62] Liao Y, Wei J, Xu Y, Zhang Z (2015). Cloning, expression and characterization of COI1 gene (AsCOI1) from Aquilaria sinensis (Lour.) Gilg. Acta Pharmaceutica Sinica B.

[63] Lu X, Jain VV, Finn PW, Perkins DL (2007). Hubs in biological interaction networks exhibit low changes in expression in experimental asthma. Molecular Systems Biology.

[64] Wang P, Lü J, Yu X (2014). Identification of important nodes in directed biological networks: A network motif approach. PloS one.

[65] Hu, J. X., Thomas, C. E. & Brunak, S. Network biology concepts in complex disease comorbidities. *Nature Reviews Genetics* (2016). 10.1038/nrg.2016.87 27498692

[66] Yan J (2009). The Arabidopsis CORONATINE INSENSITIVE1 protein is a jasmonate receptor. Plant Cell.

[67] Sheard LB (2010). Jasmonate perception by inositol-phosphate-potentiated COI1-JAZ coreceptor. Nature.

[68] Thakur, M. Biochemical and physiological inferences of elicitors in *Brassica* in inducing resistance against *Alternaria* blight (Doctoral dissertation, PAU) (2014).

[69] Chattopadhyay, C. Management of diseases of rapeseed-mustard with special reference to Indian conditions. Sustainable production of oilseeds: rapeseed mustard technology (Eds.: A. Kumar, J. S. Chauhan & C. Chattopadhyay). *Agrotech Publ. Acad. Udaipur* 364–388 (2008).

[70] Yan C, Xie D (2015). Jasmonate in plant defence: sentinel or double agent?. Plant biotechnology journal.

[71] Funahashi A, Morohashi M, Kitano H, Tanimura N (2003). CellDesigner: a process diagram editor for gene-regulatory and biochemical networks. Biosilico.

[72] Hucka M (2004). Evolving a lingua franca and associated software infrastructure for computational systems biology: the Systems Biology Markup Language (SBML) project. Systems biology.

[73] Kitano H, Funahashi A, Matsuoka Y, Oda K (2005). Using process diagrams for the graphical representation of biological networks. Nature biotechnology.

[74] Funahashi A, Jouraku A, Matsuoka Y, Kitano H (2007). Integration of CellDesigner and SABIO-RK. In silico biology.

[75] Funahashi A (2008). CellDesigner 3.5: a versatile modeling tool for biochemical networks. Proceedings of the IEEE.

[76] Dräger A (2015). SBMLsqueezer 2: context-sensitive creation of kinetic equations in biochemical networks. BMC systems biology.

[77] Dräger A, Hassis N, Supper J, Schröder A, Zell A (2008). SBMLsqueezer: a CellDesigner plug-in to generate kinetic rate equations for biochemical networks. BMC systems biology.

[78] Liebermeister W, Uhlendorf J, Klipp E (2010). Modular rate laws for enzymatic reactions: thermodynamics, elasticities and implementation. Bioinformatics.

[79] Machné R (2006). The SBML ODE Solver Library: a native API for symbolic and fast numerical analysis of reaction networks. Bioinformatics.

[80] Hoops S (2006). COPASI—a complex pathway simulator. Bioinformatics.

[81] Keller R (2013). The systems biology simulation core algorithm. BMC systems biology.

[82] Dräger A, Palsson BØ (2014). Improving collaboration by standardization efforts in systems biology. Front. Bioeng. Biotechnol.

[83] Shannon P (2003). Cytoscape: a software environment for integrated models of biomolecular interaction networks. Genome research.

[84] Zinovyev A, Viara E, Calzone L, Barillot E (2008). BiNoM: a Cytoscape plugin for manipulating and analyzing biological networks. Bioinformatics.

[85] Bonnet E (2013). BiNoM 2.0, a Cytoscape plugin for accessing and analyzing pathways using standard systems biology formats. BMC systems biology.

[86] Smoot ME, Ono K, Ruscheinski J, Wang PL, Ideker T (2011). Cytoscape 2.8: new features for data integration and network visualization. Bioinformatics.

[87] Assenov Y, Ramírez F, Schelhorn SE, Lengauer T, Albrecht M (2008). Computing topological parameters of biological networks. Bioinformatics.

[88] Doncheva NT, Assenov Y, Domingues FS, Albrecht M (2012). Topological analysis and interactive visualization of biological networks and protein structures. Nature protocols.

